# Flying Target Detection Technology Based on GNSS Multipath Signals

**DOI:** 10.3390/s24051706

**Published:** 2024-03-06

**Authors:** Pengfei Zhu, Qinglin Zhu, Xiang Dong, Mingchen Sun

**Affiliations:** China Research Institute of Radiowave Propagation, Qingdao 266107, China; zhuql1@crirp.ac.cn (Q.Z.); dongx22s@163.com (X.D.); sunmc@crirp.ac.cn (M.S.)

**Keywords:** flying target, GNSS, multipath effect, passive radar

## Abstract

In this study, a passive radar system that detects flying targets is developed in order to solve the problems associated with traditional flying target detection systems (i.e., their large size, high power consumption, complex systems, and poor battlefield survivability). On the basis of target detection, the system uses the multipath signal (which is usually eliminated as an error term in navigation and positioning), enhances it by supporting information, and utilizes the multi-source characteristics of ordinary omnidirectional global navigation satellite system (GNSS) signals. The results of a validation experiment showed that the system is able to locate a passenger airplane and obtain its flight trajectory using only one GNSS receiving antenna. The system is characterized by its light weight (less than 5 kg), low power consumption, simple system, good portability, low cost, and 24/7 and all-weather work. It can be installed in large quantities and has good prospects for development.

## 1. Introduction

Traditional flying target detection technology normally uses active radars to send ranging signals, and detects targets by analyzing the power, time delay, Doppler, and other characteristics of the echo signals [1,2]. However, this method requires the transmission of electromagnetic signals into the environment, which makes the system have a complex and bulky structure and high power consumption. In contrast, passive radars have simpler structures and lower power consumption because they do not actively transmit electromagnetic signals into the environment and only use external public signals as the signal source [3,4,5,6,7]. Additionally, passive radar systems do not require a complex structure for the signal-transmitting device, which allow them to be miniaturized and consume minimal power.

Global navigation satellite system (GNSS) signals have the advantages of global coverage, 24/7 and all-weather operation, and an open signal structure. Furthermore, in recent years, national navigation satellite systems have been developed rapidly, the number of global navigation satellites has become more abundant, and remote sensing technology using GNSS signals has become increasingly advanced. At present, this technology has realized engineering applications in the fields of sea surface altitude measurement [8,9], effective wave height measurement at sea level [10,11], the remote sensing of wind fields at sea level [12,13,14,15], the remote sensing of seawater salinity [16,17,18], and tidal detection [19,20,21]. In land surface remote sensing, numerous breakthroughs have also been made for measuring quantities such as soil moisture [22,23,24], snow thickness [25], and vegetation cover [26].

This paper develops a passive flight target detection system using GNSS signals as a signal source. The system utilizes the multipath signal, which is usually eliminated as an error term in navigation and positioning [27], as the basis for target detection, and is capable of realizing the localization of flying targets and the depiction of flight trajectories with the use of an ordinary omnidirectional GNSS antenna.

**Figure 1:**
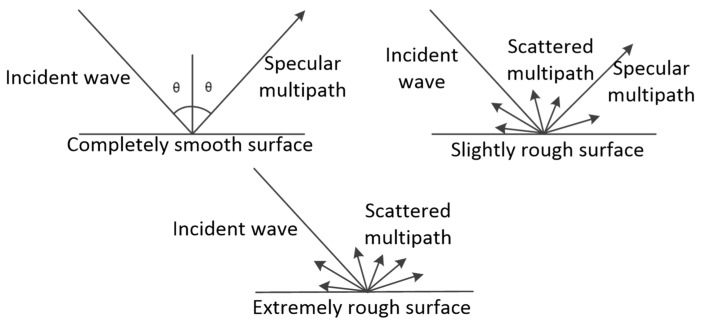
Specular and scattered multipath signals.

A multipath signal is a signal that does not reach the receiving antenna through a direct path, but enters the receiving antenna after being reflected by a reflective surface [28,29,30,31]. According to the different levels of smoothness at different positions on the reflecting surface, the multipath signal can be divided into specular multipath and scattered multipath [29,32]. As shown in Figure 1, the specular multipath signal is formed by the reflection of a smooth reflective surface. This type of signal has a more regular carrier phase and higher intensity, and is the main source of interference in multipath interference. A scattered multipath signal is a dispersed signal reflection, meaning that the signal is a cluster of different phases and a superposition of different amplitudes. Consequently, a single direction of the wave signal is weaker, and is generally considered a common noise signal. Scattered multipath signals are often manifested as low-frequency noise attached to the direct signal, and the influence on the direct signal is much smaller than that on the specular reflection multipath signal.

Conventional radar has two main methods of detecting flying targets: mechanical scanning and electrical scanning. Mechanical scanning involves the rotation of the radar antenna. Electrical scanning involves phased-array radar controls that change the direction of the radar beam by controlling the phase of the transmitted and received signals [33]. Both methods require complex system architectures and are expensive.

In contrast, the system developed in this study is a passive flying target detection system that uses GNSS signals as a signal source. The system utilizes the multipath signal (which is usually eliminated as an error term in navigation and positioning) as the basis for target detection, and utilizes the multi-source spread spectrum coding of GNSS signals and the superposition properties of specular multipath signals on direct signals to determine the position and trajectory of flying targets using an ordinary wide-beam omnidirectional GNSS antenna. The system is low-weight, easy to install, and undetectable, and consumes minimal power.

The rest of this paper is organized as follows. In Section 2.1, the model of multipath signals is presented. In Section 2.2, the detection range of GNSS signals is explained. Section 3.1 describes the experiment, and Section 3.2 discusses the results. Finally, Section 4 draws the conclusions of this study.

## 2. Materials and Methods

### 2.1. Signal Models

The reason why multipath signals have a greater impact on GNSS signals is that, unlike other noise interference signals, multipath signals have the same signal coding structure and almost the same carrier frequency as those of direct signals. Therefore, not only is the RF frontend unable to filter the multipath signal, but it also experiences difficulties in eliminating it in the signal digitization process.

**Figure 2:**
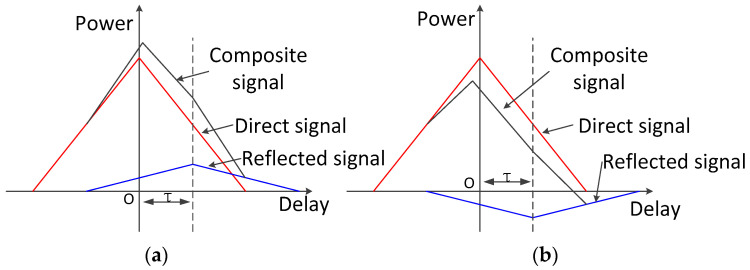
Effect of multipath signal superposition. (**a**) Superposition; (**b**) offset overlay.

The effect of the superposition of multipath signals on direct signals can be categorized into two types: enhancement superposition and offset superposition. In the case where only a single multipath signal source is considered, the superposition effect shown in Figure 2 occurs.

As indicated in Figure 2, when the multipath signal has the same phase as or a similar phase to that of the direct signal, it has an enhanced superposition effect on the direct signal. When the multipath signal and the direct signal have opposite phases, the former has an offset superposition effect on the direct signal. For the superposition effect on the receiver, changes in the reflective surface and the relative position of the navigation satellite lead to the direct channel receiving signal strength fluctuations.

In the absence of multipath effect interference, the GNSS antenna directly receives the navigation signal from the GNSS satellite. After the steps involving RF frontend filtering, down-conversion, and power amplification are processed, the signal expression is

(1) $$S_{D}\left(t\right)=\sqrt{2P_{D}}C\left(t\right)D\left(t\right)cos\left[\omega_{\;}t+\varphi_{D}\left(t\right)\right],$$

where $S_{D}$ is the signal model in the case of no multipath interference, t is the signal reception time, $P_{D}$ is the direct signal power, $C\left(t\right)$ denotes the coarse acquisition (C/A) code signal, $D\left(t\right)$ is the navigation message bit signal, ω is the carrier signal frequency, and $\varphi_{D}\left(t\right)$ is the initial carrier phase of the direct signal.

**Figure 3:**
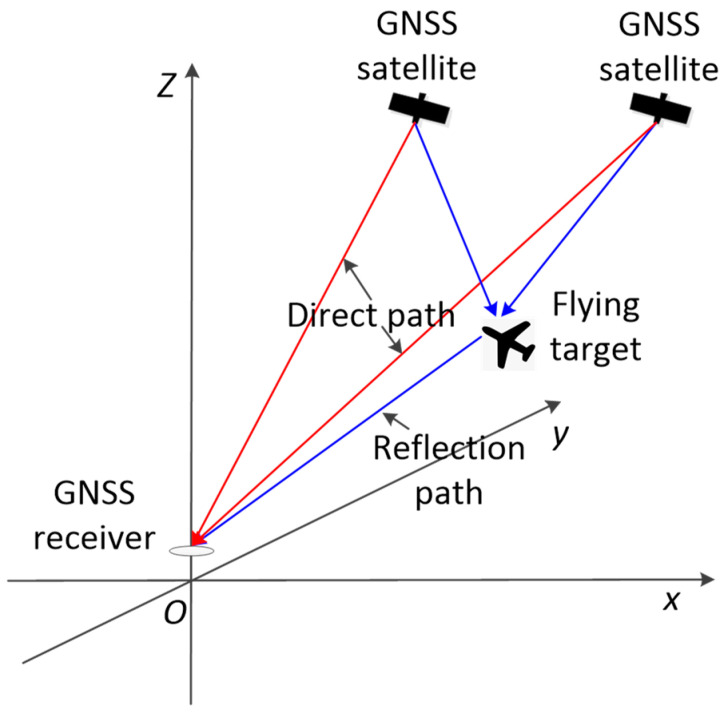
GNSS signal propagation path.

The GNSS signal propagation path after reflection from a flying target is shown in Figure 3. After being reflected by the target, part of the reflected signal is received by the GNSS receiver antenna according to the signal expression:

(2) $$S_{R}\left(t\right)=\sqrt{2P_{R}}C\left(t-\tau \right)D\left(t-\tau \right)cos\left[\omega_{\;}t+\varphi_{R}\left(t\right)\right],$$

where $P_{R}$ is the reflected signal power, τ is the time delay of the reflected signal with respect to the direct signal, and $\varphi_{D}\left(t\right)$ is the initial carrier phase of the reflected signal.

Without considering other multipath interference sources, the direct and reflected signals from the flying target are superimposed, and the signal is modeled as

(3) $$S_{\;}\left(t\right)=\sqrt{2P_{D}}C\left(t\right)D\left(t\right)cos\left[\omega_{\;}t+\varphi_{D}\left(t\right)\right]+\sqrt{2P_{R}}C\left(t-\tau \right)D\left(t-\tau \right)cos\left[\omega_{\;}t+\varphi_{R}\left(t\right)\right]+n(t)$$

where n(t) is the noise signal power.

### 2.2. Detection Range

GNSS navigation and positioning systems use spread-spectrum coding techniques that provide a favorable suppression of multipath signals with delays greater than one code slice. However, when the delay is less than one code slice, the multipath signal will distort the correlation function between the received synthesized signal and the locally generated reference signal, which leads to a code tracking error in a delay-locked loop (DLL), and the signal-to-noise ratio of the tracking result increases. This property of the multipath effect of GNSS signals is utilized in this study to monitor the direct channel signals. Furthermore, when the multipath effect generated by the flying target generates a sudden and drastic change in the signal-to-noise ratio of a satellite signal, it indicates that the path delay of the specular multipath signals generated by the flying target is less than one code slice, and the flying target enters a region of known range. This range is an ellipsoidal region, with the positions of the GNSS-receiving antenna and the navigation satellite as the foci.

**Figure 4:**
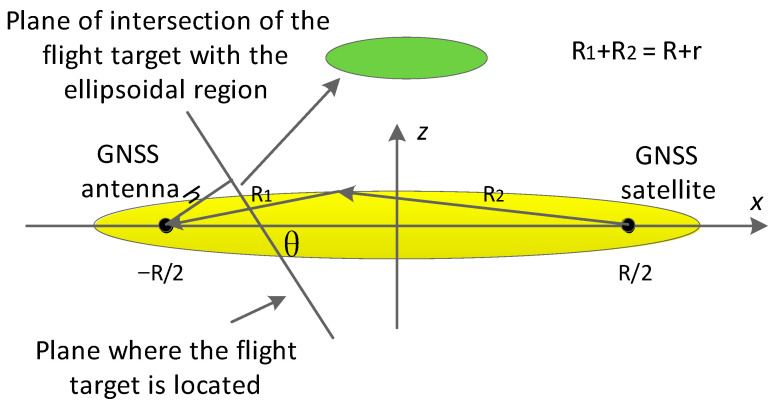
Coordinate system conversion.

The coordinate system used in this study is shown in Figure 4, in which h is the flight altitude of the flying target and θ is the satellite elevation angle. $\left(-R/2,0,0\right)$ are the coordinates of the GNSS antenna, and $\left(R/2,0,0\right)$ are the coordinates of the navigation satellite.

(4) $$\frac{x^{2}}{{\left(\frac{R}{2}\right)}^{2}}+\frac{y^{2}}{{{\left(\frac{R+r}{2}\right)}^{2}-\left(\frac{R}{2}\right)}^{2}}+\frac{z^{2}}{{{\left(\frac{R+r}{2}\right)}^{2}-\left(\frac{R}{2}\right)}^{2}}=1.$$

The expression for the plane in which the flying target is located is

(5) $$z=\frac{h}{cos\theta }-\frac{Rtan\theta }{2}-tan\theta x.$$

Inserting Equation (5) into Equation (4) enables the lengths of the long and short axes of the ellipsoid region formed by intersecting the ellipsoid at the height of the flying target to be calculated as follows:

(6) $$A=\sqrt{{\left(z_{1}-z_{2}\right)}^{2}+{\left(x_{1}-x_{2}\right)}^{2}}$$

(7) $$B=R\sqrt{\frac{R^{2}\left(2Rr+r^{2}\right)}{16}-{\left(\frac{h}{sin\theta }-\frac{R}{2}\right)}^{2}\frac{2Rr+r^{2}}{4}}$$

where $\left(x_{1},z_{1}\right)$ and $\left(x_{2},z_{2}\right)$ are the coordinates of the two intersections between the long axis of the ellipse and the ellipsoid region.

**Figure 5:**
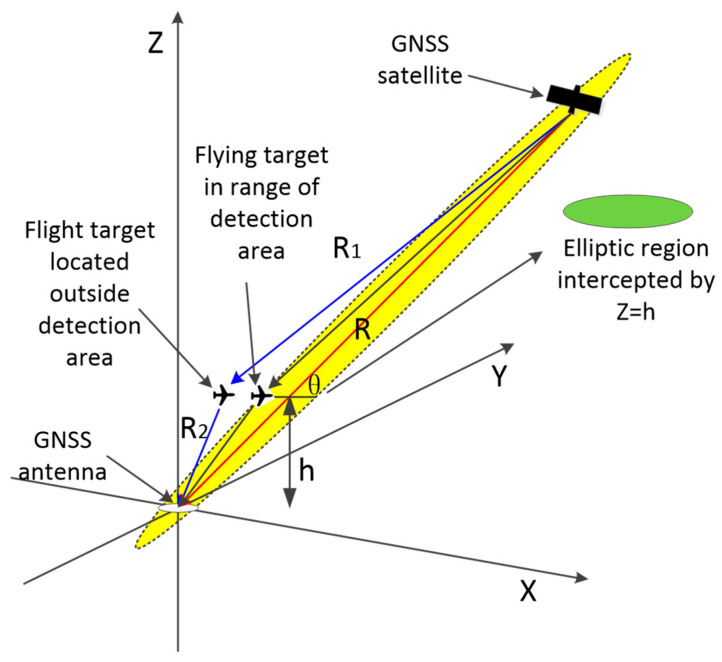
GNSS signal propagation model. The elliptical region represents the multipath detection area.

In order to describe the range of the detection area more intuitively, the radar system was set up in accordance with the station center coordinate system. As shown in Figure 5, the coordinate system features the GNSS-receiving antenna as the origin, the zenith direction as the Z-axis, the due-east direction as the X-axis, and the due-north direction as the Y-axis, where R is the distance between the navigation satellite and the GNSS-receiving antenna, $R_{1}$ is the distance between the navigation satellite and the flying target, $R_{2}$ is the distance between the flying target and the GNSS-receiving antenna, and r is the transmission distance of a code slice time signal. When the position of the flying target satisfies the condition $R_{1}+R_{2}<R+r$, it enters the detection area (i.e., the yellow ellipsoidal region in the figure).

In the ellipsoid region, the GNSS antenna and navigation satellite are the two foci of the ellipsoid, h is the height of the flying target, and θ is the satellite elevation angle.

The upper and lower limits of the azimuth are, respectively,

(8) $$\varphi_{a}=\varphi +∆\varphi =\varphi +\tan^{-1}\left(\frac{B}{2hcot\theta }\right)$$

(9) $$\varphi_{u}=\varphi -∆\varphi =\varphi -\tan^{-1}\left(\frac{a}{2hcot\theta }\right)$$

where *B* is the short axis length, ∆φ is the maximum change in azimuth, and φ is the navigation satellite azimuth.

The upper and lower elevation limits are, respectively,

(10) $$\emptyset_{a}=\theta +\tan^{-1}\left(\frac{z_{1}}{x_{1}+\frac{R}{2}}\right)$$

(11) $$\emptyset_{a}=\theta +\tan^{-1}\left(\frac{z_{2}}{x_{2}+\frac{R}{2}}\right)$$

where $\left(x_{1},z_{1}\right)$ and $\left(x_{2},z_{2}\right)$ are the coordinates of the two intersections of the long axis of the ellipse with the ellipsoid region, and θ is the elevation angle of the navigation satellite.

**Figure 6:**
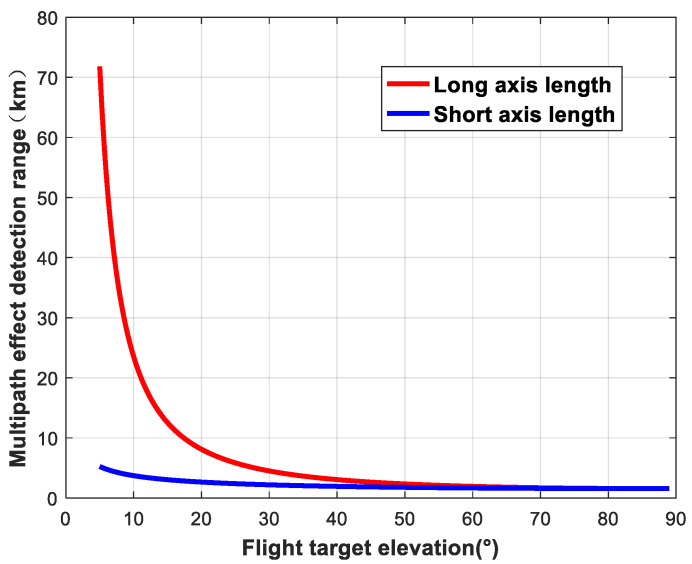
Variation in detection area as a function of satellite elevation (major and minor axes).

**Figure 7:**
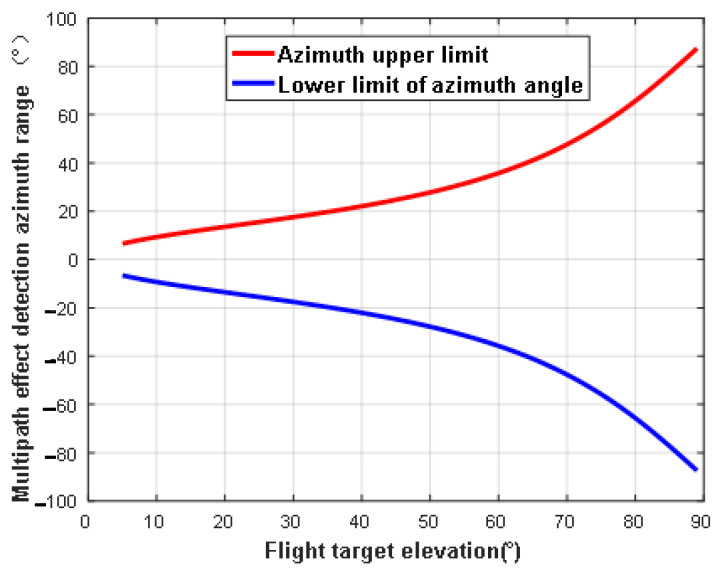
Variation in detection area change as a function of satellite elevation (azimuth).

**Figure 8:**
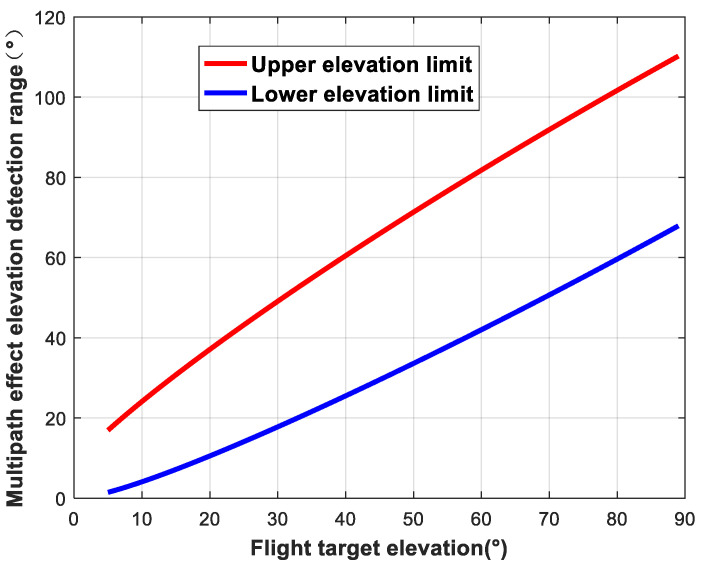
Variation in detection area as a function of satellite elevation (elevation).

The size of the ellipsoidal region formed by the intersection between the plane where the flying target is located and the ellipsoidal region is related to the satellite elevation angle and the height of the flying target. When the altitude of the flying target is fixed, the change in the detection area as a function of the elevation angle of the navigation satellite can be analyzed. We set the star–ground distance to *R* = 25,000 km and the flying target altitude to *h* = 1 km. The variations in the detection area of ellipsoids are shown in Figure 6, Figure 7 and Figure 8.

**Figure 9:**
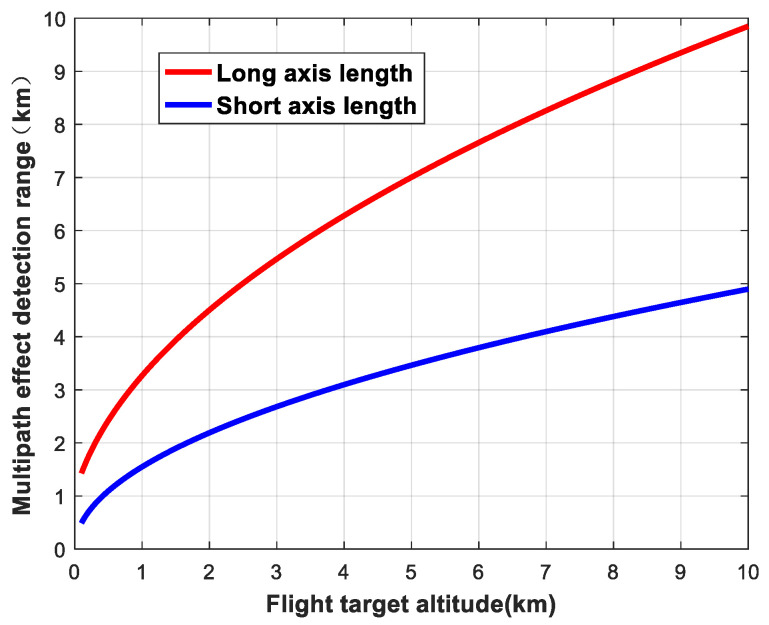
Variation in detection area as a function of flying target altitude (long and short axes).

**Figure 10:**
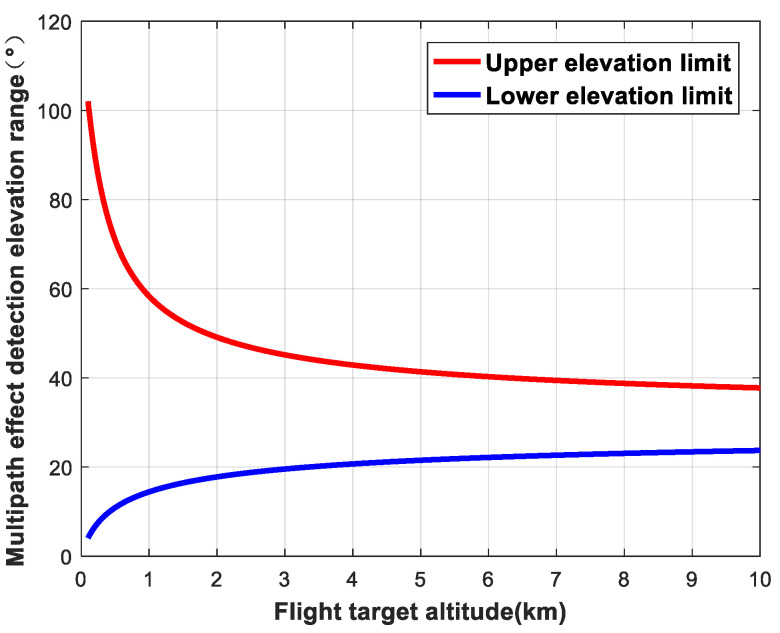
Variation in detection area as a function of flying target altitude (elevation).

**Figure 11:**
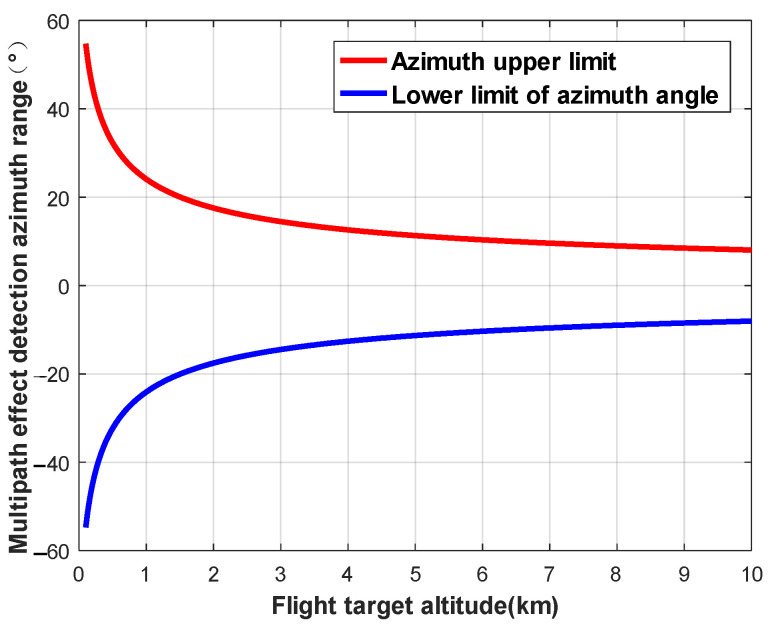
Variation in detection area as a function of flying target altitude (azimuth).

When the elevation angle of the navigation satellite is fixed, the change in the detection area as a function of the altitude of the flying target can be analyzed. We set the star–ground distance to *R* = 25,000 km and the elevation angle of the navigation satellite to 30°. The variations in the detection area of ellipsoids are shown in Figure 9, Figure 10 and Figure 11.

## 3. Results

### 3.1. Experiment

**Figure 12:**
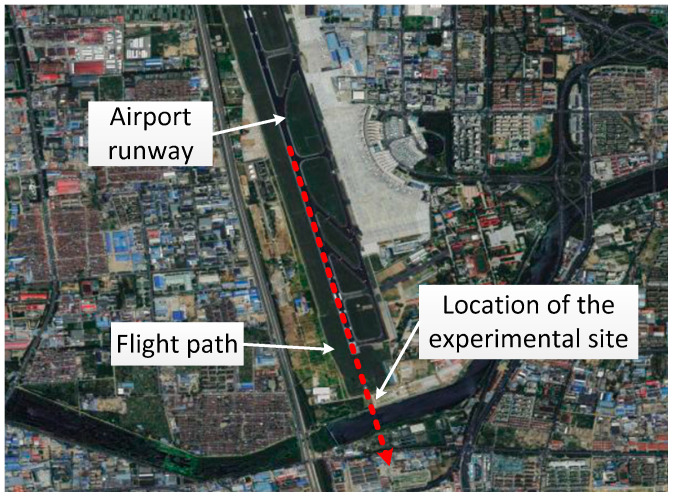
Aerial view of the airport runway, flight path, and site of the experiment.

In order to verify the feasibility of the method, a passenger aircraft departing from Qingdao Liuting Airport was detected using signal receiving equipment. Qingdao Liuting Airport is located in Chengyang District of Qingdao City, is a class 4E civil international airport, and is one of the twelve major trunk airports in China. There is one main runway at the airport, and passenger airplanes usually take off in the southeast direction. The airport runway, flight path, and site of the experiment are shown in Figure 12.

First, the test equipment was built at the southeast end of the runway. The experimental site was open. The experimental equipment was arranged as follows:

1. The antenna was set up in an open position facing the zenith direction;
2. The IF signal collector and signal-processing host were placed within a certain range of the antenna support;
3. The IF signal collector was connected to the PC computer cable, and the GNSS signal-receiving antenna was connected to the IF signal collector through the RF cable;
4. The video-recording equipment was set up to record the sky in the zenith direction.

The devices used in the experiment are shown in Table 1.

**Figure 13:**
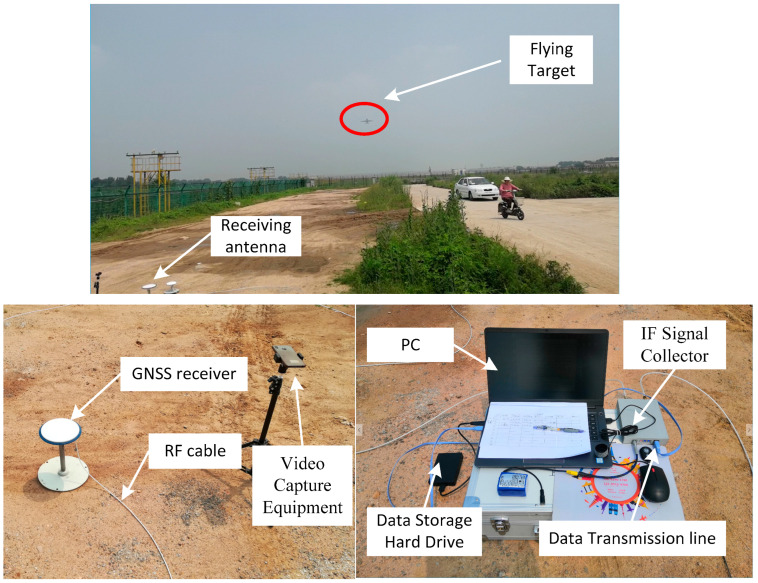
Site of the experiment and experimental setup.

The site of the experiment and the experimental setup are shown in Figure 13.

After the test equipment was built, the host computer was turned on, the signal acquisition software was initialized, and the IF data storage location was selected. 

After the flying target flew over the receiving antenna, the appearance, passing vertex, and departure time of the flying target were recorded. Afterwards, the recorded results were compared with the video to ensure their accuracy. The information recorded during the experiment is displayed in Table 2.

### 3.2. Analysis of Results

**Figure 14:**
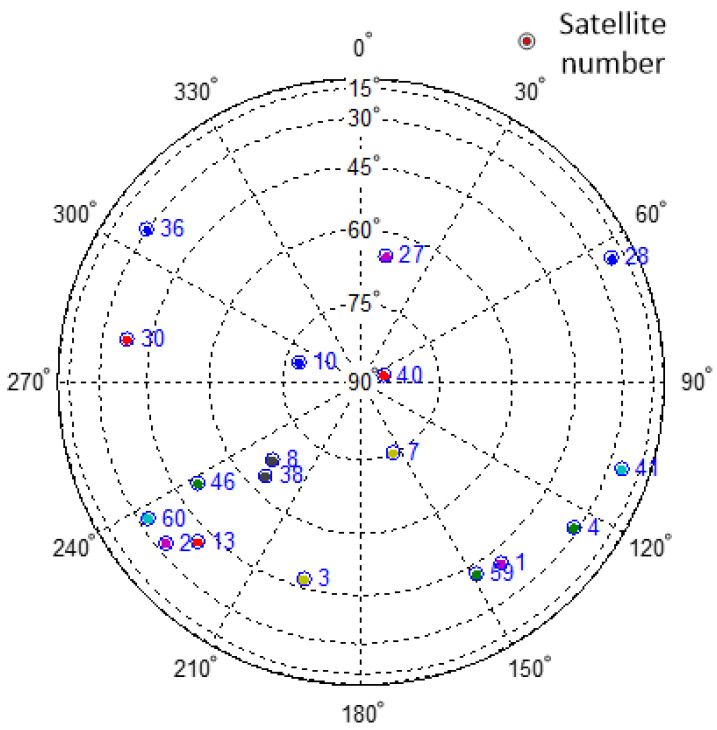
Starry sky map. Different colored dots represent different satellites.

After the experiment was completed, the intermediate frequency data were captured and tracked, the coordinates of the experimental site and navigation satellite were calculated, and a starry sky map of the site was drawn, as shown in Figure 14. 

**Figure 15:**
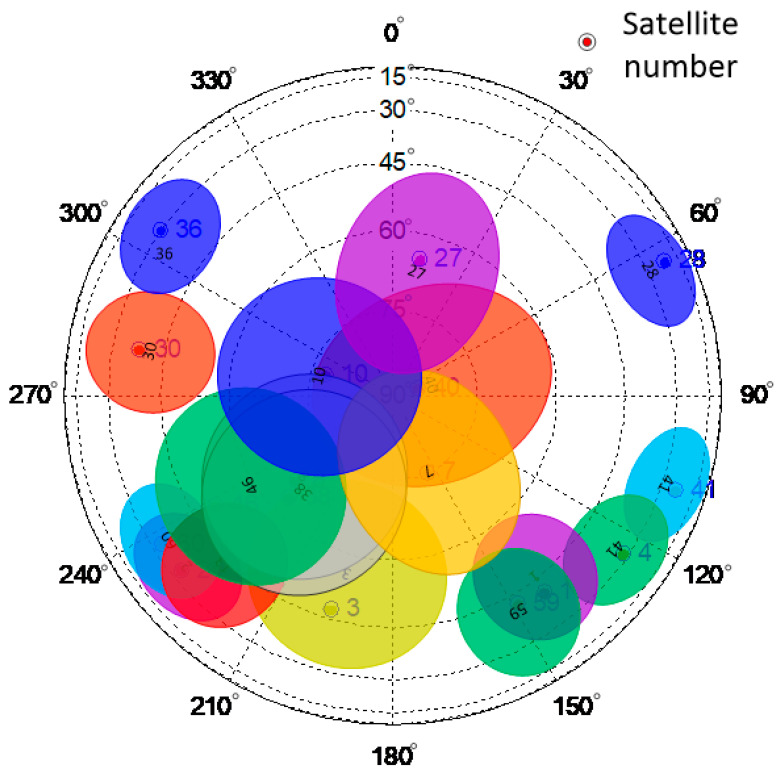
Schematic diagram of detection area for each GNSS satellite. Different colored dots and ovals represent different satellites and the multipath detection range of the satellite.

In Figure 14, the spokes represent different azimuth angles, the concentric circles represent different elevations, and the solid dots represent the locations of the GNSS satellites. The detection ranges of different satellite signals are based on the satellite elevation and azimuth angles, as shown in Figure 15.

**Figure 16:**
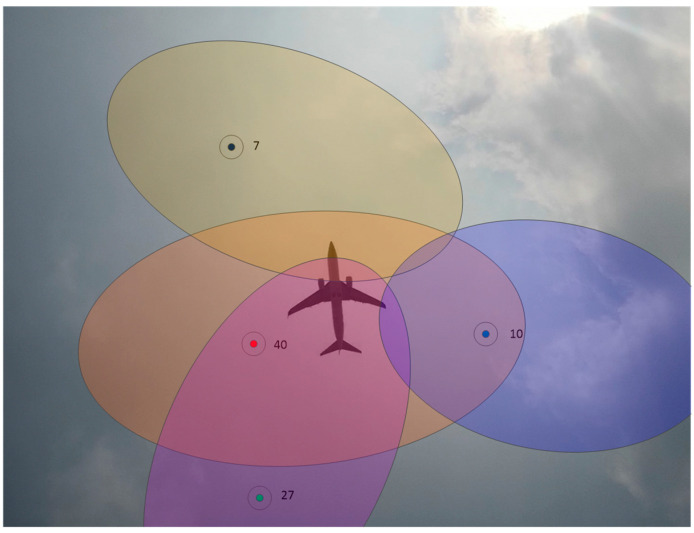
Starry sky map of the experiment as the flying target passed over the GNSS antenna’s zenith. Different colored dots and ovals represent different satellites and the multipath detection range of the satellite.

In Figure 15, the elliptical areas with different colors correspond to the detection ranges of the navigation satellites. In the experiment, when the flying target passed over the GNSS antenna’s zenith, the estimated positions of the satellites present at the time were determined, as shown in Figure 16.

**Figure 17:**
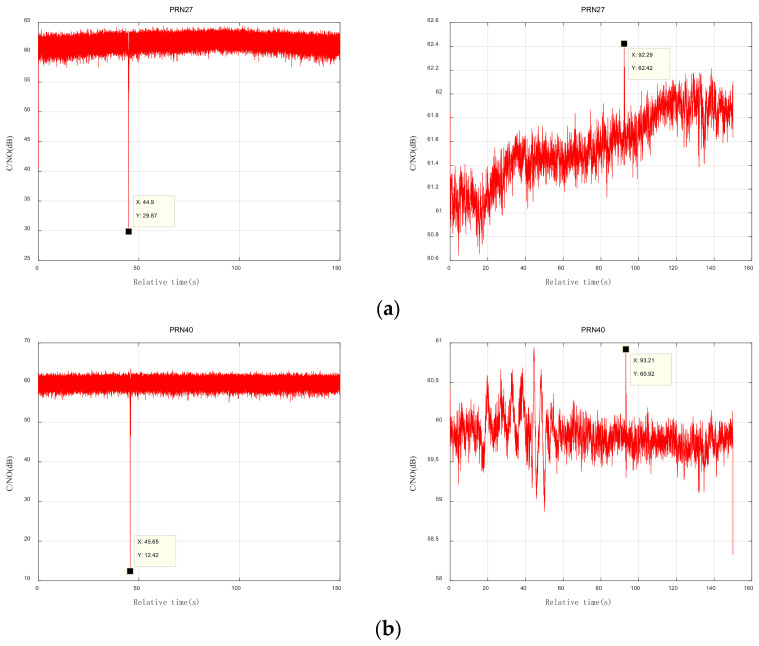

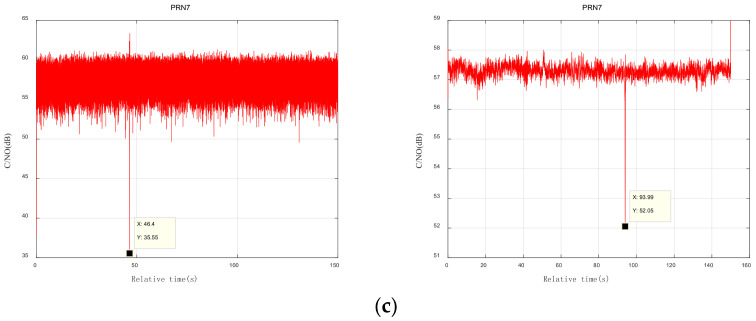
Carrier-to-noise ratio calculations. (**a**) Variation in signal-to-noise ratio of satellite 27 signals; (**b**) variation in signal-to-noise ratio of satellite 40 signals; (**c**) variation in signal-to-noise ratio of satellite 7 signals.

The changes in the signal-to-noise ratios of different satellites were used to determine the satellite timing sequence of the flying target as it crossed the detection range of different satellites, and the trajectory of the flying target could be obtained as a result. As shown in Figure 17.

**Figure 18:**
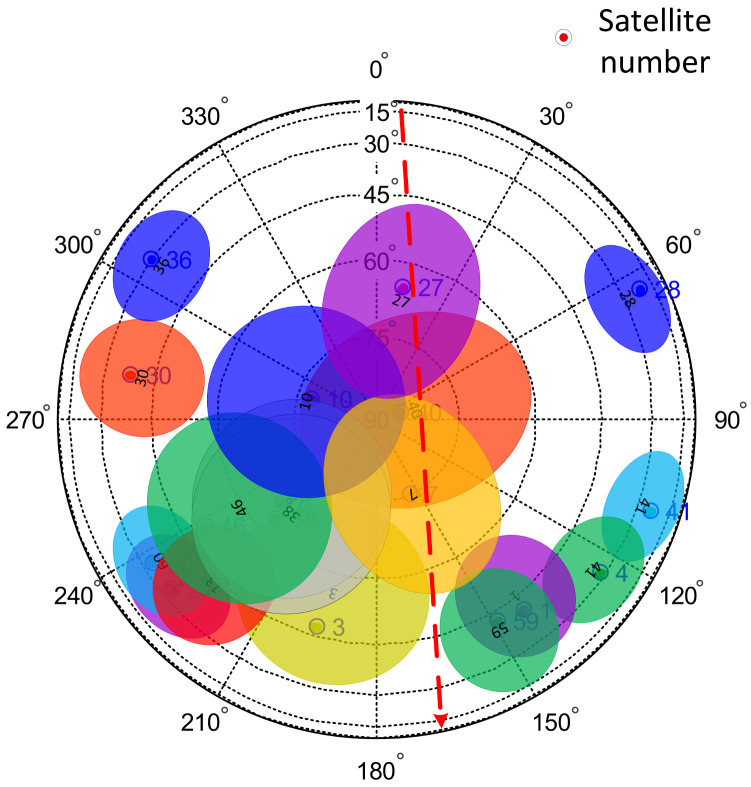
Estimation of the trajectory of the flying target. The red dashed arrow represents the direction of flight of the flying target, and different colored dots and ovals represent different satellites and the multipath detection range of the satellite.

In the first experiment, according to the carrier-to-noise ratio data, when the relative times were 44.9 s, 45.65 s, and 46.4 s, the flying target passed through the multipath detection areas of satellites 27, 40, and 7, respectively. In the second experiment, according to the carrier-to-noise ratio data, when the relative times were 92.29 s, 93.21 s, and 93.99 s, the flying target passed through the multipath detection areas of satellites 27, 40, and 7, respectively. Using this information, the trajectory of the flying target was drawn, as shown in Figure 18. The trajectory of the flying target was consistent with the actual flight path of the flying target shown in Figure 12.

## 4. Conclusions

In this study, we described in detail the technical principles of GNSS-based multipath signals for the detection of flying targets, and we conducted an experiment at Qingdao Liuting Airport to verify the feasibility of the technical solution. The results demonstrate that the use of passive radar technology enables the determination of trajectories of targets that are difficult to detect; it also enables low-cost systems with structural simplicity, portability, and minimal power consumption to be utilized. In recent years, the increasing number of small aircrafts, such as drones, gliders, and small helicopters, has added to the difficulties of the safety management of urban airspace and other important airspaces. Traditional small aircraft detection methods mainly include radar detection, photoelectric detection, acoustic wave detection, etc. However, radar systems are complex, large, power-hungry and costly, optoelectronic devices are greatly affected by weather, and acoustic devices are susceptible to interference and have poor accuracy. In follow-up work, we hope to be able to apply this technology to the detection and management of small aircrafts. Additionally, because the hardware environment is not currently available for the real-time detection of flying targets, the main aim of future work will be to build a hardware system that detects flying targets in real time.

## Figures and Tables

**Table 1:** Devices used in the experiment.

Num.	Device	Quantity
1	GNSS receiver antenna	1
2	Antenna bracket	1
3	RF cable	1
4	Video capture equipment	1
5	IF signal collector	1
6	Data storage hard drive	1
7	Data processing terminal	1

**Table 2:** Data recorded during the experiment.

Num.	Event	Absolute Time	Relative Time (s)
1	Start Collection	10:14:00	0
2	Target Appearance	10:14:41	41
3	Target Over Top	10:14:51	51
4	Target Leaving	10:15:12	72
5	Stop Collection	10:16:30	150
6	Start Collection	10:16:30	0
7	Target Appearance	10:17:59	89
8	Target Over Top	10:18:08	98
9	Target Leaving	10:18:27	117
10	Stop Collection	10:19:00	150

## Data Availability

The data set is available on request to the corresponding authors.
